# A Comprehensive Study of the Quality of Fat-Tailed Sheep Carcasses in Greece

**DOI:** 10.3390/ani12151998

**Published:** 2022-08-07

**Authors:** Angeliki Argyriadou, Anestis Tsitsos, Ioanna Stylianaki, Sotiria Vouraki, Theodoros Kallitsis, Vangelis Economou, Georgios Arsenos

**Affiliations:** 1 Laboratory of Animal Husbandry, School of Veterinary Medicine, Faculty of Health Sciences, Aristotle University, 54124 Thessaloniki, Greece; 2 Laboratory of Hygiene of Food of Animal Origin—Veterinary Public Health, School of Veterinary Medicine, Faculty of Health Sciences, Aristotle University, 54124 Thessaloniki, Greece; 3 Laboratory of Pathology, School of Veterinary Medicine, Faculty of Health Sciences, Aristotle University, 54124 Thessaloniki, Greece

**Keywords:** fat-tailed sheep, thin-tailed sheep, carcass, meat quality, dairy sheep

## Abstract

**Simple Summary:**

Sheep meat in Greece is considered a by-product of dairy production, associated with undefined quality and low revenues. Production is based on slaughtering of young lambs and consists of light carcasses. Heavy carcasses are scarce and mainly derived from rams and ewes that are no longer appropriate for dairy farming due to age, health or productivity issues; no dietary treatments or fattening protocols are implemented prior to slaughter. Improving production sustainability implies more efficient use of available resources, including local fat-tailed sheep that are reared in high numbers for their milk, although their carcasses are undervalued; supportive research data are scarce. Hence, we used objective methods to assess the quality of carcasses of fat-tailed sheep slaughtered at different live weights (representing five developmental stages, from young lambs to adult sheep) and to compare them with carcasses of thin-tailed sheep, which are considered superior. We found that quality of fat-tailed sheep carcasses was equal or higher compared to thin-tailed. Differences between sexes and developmental stages existed. Slaughtering of fat-tailed sheep at higher live weights (50–70% of the average adult sheep weight) will benefit production quality and quantity, improving profitability and sustainability of the sector.

**Abstract:**

Sheep farming in Greece is focused on milk production. Meat is considered a by-product and consists mainly light carcasses of undefined quality. The main challenge of the sector is to ensure sustainability, and hence efforts are towards efficient use of available resources, including undervalued carcasses of local fat-tailed sheep. The objective here was twofold: (i) to assess the carcass quality of fat-tailed sheep slaughtered at different live weights and (ii) to compare them with carcasses from thin-tailed sheep. In total, 146 fat-tailed and 97 thin-tailed dairy sheep were used. They belonged to five live-weight categories (LWC), representing 25%, 35%, 50%, 70% and 100% of mature body weight. Carcass length/weight/yield/pH and wither height were recorded. Muscle fiber minimum Feret’s diameter and meat color/tenderness/moisture/lipid and protein content were determined. Sex and LWC differences in fat-tailed sheep were assessed. Parametric and non-parametric tests were used to compare with thin-tailed sheep, considering the effects of LWC, sex and their interactions with sheep population (fat-tailed/thin-tailed). Most traits were significantly different (*p* < 0.05) between groups of fat-tailed sheep. Carcass yield of fat-tailed sheep was significantly higher compared to thin-tailed (*p* < 0.01). Interactions of sheep population with LWC or sex affected wither height, carcass pH, meat color and tenderness (*p* < 0.05). Fat-tailed sheep meat quality is equal or higher compared to thin-tailed. Finishing weights corresponding to 50 and 70% LWC may improve capitalization of fat-tailed carcasses.

## 1. Introduction

Sheep meat production in Greece comprises more than one-fourth (27.2%) of domestic non-poultry meat production [1] and one-tenth (11.7%) of total EU sheep meat production. Despite the size of the sector, sheep meat in Greece is considered a by-product of dairy production associated with very low revenues [2]. Most slaughtered animals are young lambs (approximately one to three months old). Other categories include ewes and rams removed from production due to age or health problems without any specific dietary treatment prior to slaughter. Hence, the quality of their carcasses is poor, with large variation regarding classification. In practice, such carcasses are sold whole or in halves. Smaller cuts and marketable sheep meat products are scarce. Hence, consumption is mostly seasonal and related to religious or festive occasions [3,4]. 

In Greece, consumption of sheep meat shows a constant decline and is being replaced by other types of meat (bovine, swine, poultry), for which Greece is less self-sufficient and their production is less sustainable [5]. One of the major goals of the EU agricultural outlook for the next decade is to achieve more efficient production systems [6]. Regarding meat production in Greece, the notion is that efficient use of available resources will increase self-sufficiency and limit imports. In this context, production of heavier sheep carcasses will allow year-round market supply and will increase the marketability of sheep meat [2,7]. However, production of heavier sheep carcasses requires implementation of special management practices and designated nutrition, considering the large variation in breeds and their crosses comprising the national flock in Greece that is oriented towards milk production. Moreover, the national flock involves fat-tailed dairy sheep in significant numbers (mostly animals of Chios and Lesvos breeds) that show remarkable resilience in challenging habitats in terms of food availability and temperature fluctuations [8], but their meat production is generally undervalued. 

To achieve efficient use and better capitalization of sheep meat production in Greece, it is necessary to define the baseline quality of produced carcasses, without any dietary or other interventions. Special attention should be drawn to fat-tailed sheep carcasses, which are largely considered of lower quality compared to thin-tailed, a perception that affects their marketability, despite the scarcity of supportive scientific evidence. Investigation of possible differences between the two sheep populations of the national flock, fat-tailed and thin-tailed, also considering sex and weight variation, will highlight the strong and weak points of meat production in each case and reveal potential for improvement through the implementation of customized management practices. In this study our objective was twofold: (i) to characterize and assess the carcass quality of fat-tailed sheep reared in Greece and slaughtered at different live weights and (ii) to perform a comparison with carcasses of thin-tailed sheep.

## 2. Materials and Methods

### 2.1. Animals and Study Design

A total of 146 fat-tailed and 97 thin-tailed dairy sheep of both sexes were used. Animals were selected with the aim of better representing the sheep populations reared in Greece. Fat-tailed sheep were mainly Chios purebreds or their crosses, whereas thin-tailed sheep were crossbreds of the various thin-tailed dairy sheep breeds that are common in Greece. The population of the latter is greatly diverse, with undefined genetic background; although in some Greek dairy sheep farms, thin-tailed sheep of specific breeds are present or even exclusively bred, most farms use sheep of more than one breed and their crosses. The selected animals came from semi-intensively reared flocks, which represent the most common sheep farming system in Greece; sheep were grazing daily during spring and summer months in natural grasslands, and their diet also included roughage (wheat straw and alfalfa hay) and a concentrate mix of 16% total protein content, consisting mainly of soya and corn. They were slaughtered at different live weights and allocated into five different live-weight categories (LWC), representing 25%, 35%, 50%, 70% and 100% of mature weight, which ranges approximately from 55–80 kg, considering both sexes (Table 1).

Slaughtering took place in two approved commercial abattoirs in Greece, according to EU standards [9]. Prior to slaughter, all animals were weighted (live weight), and their wither height was measured. Carcasses were commercially dressed; light carcasses (25%, 35% and 50% of mature weight) included head and viscera (heart, lungs, liver, internal fat and kidneys). Following carcass dressing and prior to refrigeration, hot carcass weight was recorded; then, carcass yield was calculated as the ratio of carcass weight to live weight. With carcasses suspended by the hind legs, carcass length from the base of the tail to the base of the neck was measured [10]. One hour after refrigeration, carcass pH was measured non-destructively, using a portable pH meter (FiveGo pH meter F2, Mettler Toledo, Zaventem, Belgium), on the medial side of the right leg, following muscle incision. The average of two consecutive measurements on the same point of incision was calculated. Prior to use, the instrument was calibrated following the manufacturer’s instructions, with two buffer solutions of standard pH, 4.00 and 7.00.

### 2.2. Sample Collection

Samples of randomly selected carcasses from each LWC and sex were obtained for further analysis. Specifically, samples from a standardized area of the psoas major muscle were collected immediately after slaughter, for histological and morphometrical evaluation. In total, 76 samples were collected, 34 of which were from fat-tailed sheep. Furthermore, approximately 24 h after slaughter, meat samples 1–2 cm thick were obtained from the 13th rib of the left side of cold carcasses and from a standardized area of the quadriceps muscle in the left hind leg. A total of 99 meat samples from the 13th rib (58 of which were obtained from fat-tailed sheep carcasses) were vacuum-packed and stored at 4 °C for six days until they were subjected to quality assessment. Last, meat samples from the thigh region (*M. quadriceps femoris*), 43 in total (27 of which were from fat-tailed sheep carcasses) and approximately 100 g each, were vacuum-packed and stored at −20 °C until they were used for physicochemical analyses.

### 2.3. Muscle Histomorphometry

Formalin-fixed psoas major muscle samples were embedded in paraffin, cut at 4 μm, and stained with hematoxylin and eosin. For morphometry, fifteen 10× magnification muscle cross-section images were captured from each sample. Then, two to eight images were randomly selected for morphometrical analysis; a total of 1001 to 1766 muscle fibers per animal were analyzed. The muscle fiber minimum Feret’s diameter, which represents a geometrical parameter that remains largely unaffected by miscalculations due to orientation and sectioning angles [11], was automatically calculated in each image. Morphometry was performed using the “analyze particles” command of the ImageJ 1.53k software (NIH, Bethesda, MD, USA) based on a previously described methodology [12].

### 2.4. Meat Quality Assessment

Quality assessment of meat samples from the 13th rib included texture profile analysis (TPA), pH measurement and colorimetry. Meat color parameters (lightness—L*, redness—a*, yellowness—b*) were measured on freshly exposed meat samples directly after unpacking, using a colorimeter (CR-410 Chroma meter, Konica Minolta, New Jersey, USA) with 50 mm aperture size, illuminant C and 2° observer. Prior to scanning, calibration of the colorimeter was performed according to the manufacturer’s instructions with a white tile of standard parameters (Y: 94.8/X: 0.3130/y: 0.3190). Three consecutive scans perpendicular to the myofibrils were performed at the same position on each sample; between scans, the measuring head of the colorimeter was rotated 90 degrees clockwise. Measured values of L*, a* and b* were averaged over the three scans of each sample, and the mean values were considered for statistical analyses. Mean a* and b* values of each sample were used to calculate chroma and hue angle according to the following formulae, as described by the American Meat Society Association [13]:

Chroma (saturation index) = (a*^2^ + b*^2^)^1/2^ (1)

(2) $$\mathrm{Hue}\;\mathrm{angle}=\mathrm{arctangent}(\frac{b^{*}}{a^{*}})$$

Meat pH measurement and TPA were performed as described previously by [14]. Briefly, a portable pH meter (FiveGo pH meter F2, Mettler Toledo, Zaventem, Belgium) was used. The average value of two consecutive measurements on the same point of each sample was used for statistical analyses. A Stable Micro Systems TA.HD plus Texture Analyser with a flat-faced cylindrical 1.27 cm diameter probe and the Exponent software (version 6.1.16.0, Stable Micro Systems Ltd., Surrey, UK) were used for TPA. Oval-shaped pieces (1–2 cm thick) were extracted from the center of meat samples and used in a double-compression cycle test. Probe pre-test speed of 1.00 mm/s and test and post-test speed of 5.00 mm/s were implemented, achieving a 40% deformation in each cycle. Time between cycles was 2.02 s. The calculated parameters included “Hardness 1”, “Hardness 2”, “Cohesiveness”, “Springiness” and “Chewiness” as previously described by Skaperda et al. [14]. The first two represented the hardness of meat samples during the first and second bite, respectively. Cohesiveness and springiness indicated resistance to deformation and capacity of recovering after deformation, respectively, whereas chewiness represented the energy required to chew samples until they can be swallowed [15,16].

### 2.5. Physicochemical Properties of Meat

Physicochemical analyses of meat samples from the thigh region included measurements of total lipid, protein and moisture content. Samples were first comminuted with a Warring laboratory blender. For the estimation of moisture content, 3 g of meat were placed on an aluminum pan and analyzed at 105 °C with a dedicated moisture analyzer (MB27, Ohaus, Parsippany, NJ, USA), according to the manufacturer’s instructions. Total lipid content was estimated with the Weibull–Stoldt method, with hydrolysis as the first step and extraction in Soxtherm, according to AOAC method 991.36. In brief, a meat sample of 10 g was placed into a beaker. The protein of the sample was digested with boiling hydrochloric acid (4 mol/L) in order to break the lipo-protein bonds. Immediately after hydrolysis, the digestion mixture was filtrated with a pleated filter moistened with water. After drying, the remaining fat was extracted from the filter with petroleum ether of 40 to 60 °C boiling range. Following solvent evaporation, the samples were dried and weighed. Total lipid content was calculated based on the difference between the initial sample weight and the weight at the end of analysis. Total protein was determined according to AOAC Official Method 928.08. In brief, 1.5 g of meat was placed in digestion tubes. The organically bound nitrogen of the sample was digested at 400 °C, with concentrated sulphuric acid and a catalyst, and broken down to ammonium sulphate. After the addition of 32% sodium hydroxide, ammonia was released by water stream distillation and trapped in a 2% solution of boric acid. The solution was then titrated against a 0.1 mL/L hydrochloric acid solution. The total protein content of the sample was calculated by multiplying the total nitrogen content by 6.25.

### 2.6. Statistical Analyses

Data were analyzed with R programming language (software version 4.1.2, R core team, Vienna, Austria) [17]. Descriptive statistics were calculated using “psych” and “dplyr” statistical packages [18,19]. Regarding body (live weight, wither height, muscle fiber minimum Feret’s diameter), carcass (hot carcass weight, carcass yield/length/pH) and meat quality traits (meat pH/moisture/protein content/lipid content, TPA parameters—hardness 1, hardness 2, springiness, cohesiveness, chewiness—and meat color traits—L*, a*, b*, chroma, hue angle) of fat-tailed sheep, differences between sexes and LWCs were assessed with non-parametric Mann–Whitney U tests and Kruskal–Wallis one-way analyses of variance, respectively. Following Kruskal–Wallis analyses, Dunn’s tests with Bonferroni correction were used to detect statistically significant differences between groups. The latter analyses were not performed on muscle fiber minimum Feret’s diameter due to the limited number of observations in some LWCs of fat-tailed sheep (less than four per LWC). 

Mann–Whitney U tests were also performed to compare fat-tailed and thin-tailed sheep concerning the above traits. The effect of sheep population (fat-tailed or thin-tailed) on the above traits, when accounting also for the effects of LWC or sex and their interaction with sheep population, was assessed with two-way analyses of variance (ANOVA). The models for the latter analyses were of the following general form:

Y_ghi_ = μ + SP_g_ + LS_h_ + SP_g_LS_h_ + e_ghi_, (3)

where Y_ghi_ is the dependent variable (one of the body, carcass or meat quality traits, as presented above), μ is the overall population mean, SP_g_ is the fixed effect of sheep population (2 levels: fat-tailed, thin-tailed), LS_h_ is the fixed effect of LWC (5 levels: 25, 35, 50, 70, 100% of mature weight) or sex (2 levels: male, female), SP_g_LS_h_ is the fixed effect of the interaction between sheep population and LWC or sex, and e_ghi_ is the residual error. 

Tukey’s range tests were performed for post hoc testing to allow for pairwise comparisons between groups. ANOVA and Tukey’s tests including LWC and the interaction effects were not performed on meat moisture, protein content, lipid content and muscle fiber minimum Feret’s diameter, due to lack of observations (less than or equal to 1) in some groups. Homogeneity of variances and normality of distributions were assessed with Levene’s test and Kolmogorov–Smirnov test, respectively (*p* > 0.05). Residuals versus fitted and Q–Q plots of the analyses were considered, as well. In the case that the assumptions of homoscedasticity and normality of ANOVA residuals were not met, a non-parametric equivalent Scheirer–Ray–Hare test was performed. Specifically, the effects of LWC, sheep population and their interaction on live weight, carcass length, hot carcass weight, carcass yield, carcass pH, L*, a*, chroma, meat pH and cohesiveness were assessed with Scheirer–Ray–Hare tests. The same applied for the effects of sex, sheep population and their interaction on live weight, carcass length, hot carcass weight, carcass yield, carcass pH, muscle fiber minimum Feret’s diameter, meat pH, hardness 1 and 2, chewiness, moisture and lipid and protein content. Statistical packages “stats” and “rcompanion” [17,20] were used for the above analyses, and level of statistical significance was set at *p* = 0.05.

## 3. Results

### 3.1. Descriptive Statistics and Quality Assessment of Fat-Tailed Sheep Carcasses

**Figure 1:**
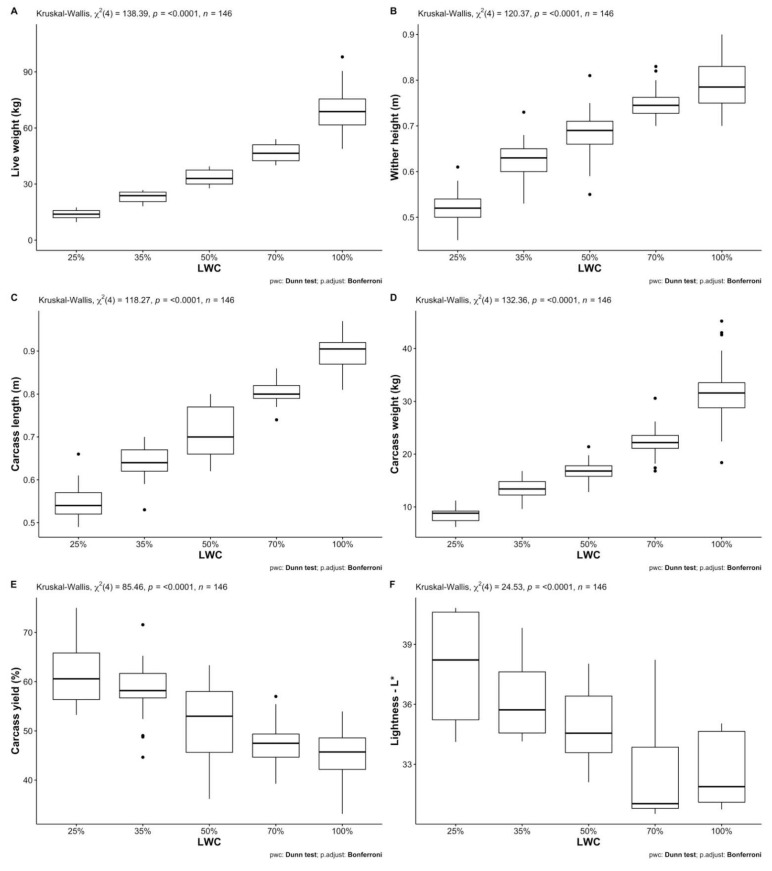

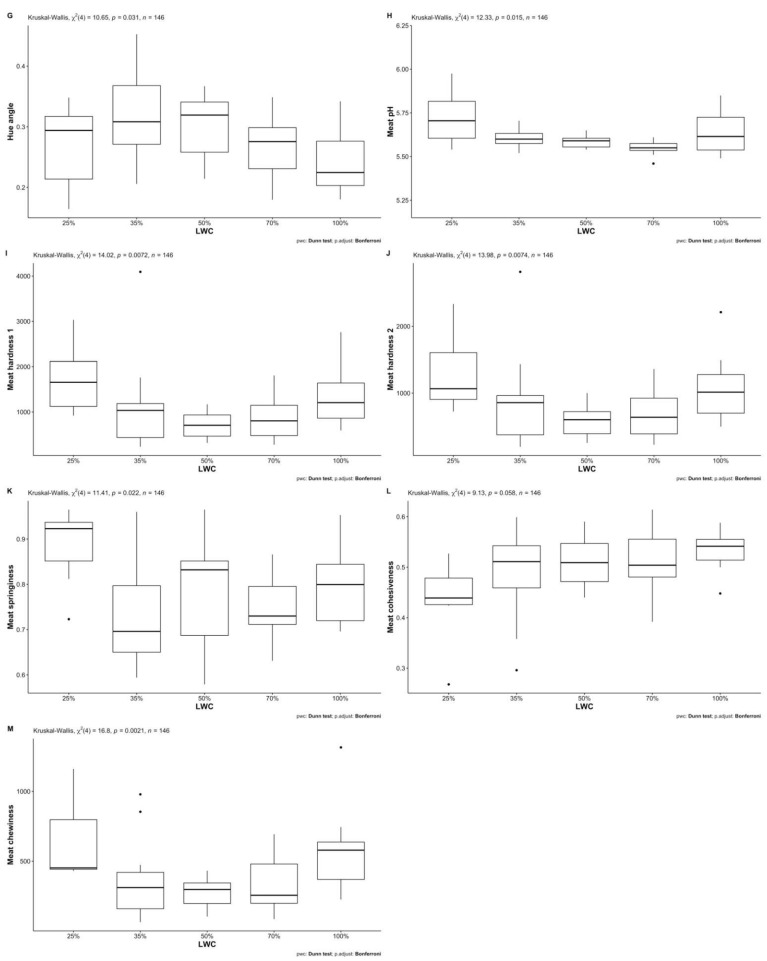
Dunn’s post hoc analysis for pairwise comparisons between live-weight categories (LWC) of fat-tailed sheep regarding the tested traits: (**A**) Live weight—in kg; (**B**) Wither height—in m; (**C**) Carcass length—in m; (**D**) Carcass weight—in kg; (**E**) Carcass yield—as %; (**F**) Lightness—L*; (**G**) Hue angle; (**H**) Meat pH; (**I**) Meat hardness 1; (**J**) Meat hardness 2; (**K**) Meat springiness; (**L**) Meat cohesiveness; (**M**) Meat chewiness.

Descriptive statistics of all studied traits for both fat-tailed and thin-tailed sheep per LWC and sex are presented in Table 2 (a and b, respectively). Mann–Whitney U tests revealed statistically significant differences between female and male fat-tailed sheep regarding live weight (W = 3184, *p* < 0.05), carcass yield (W = 1803, *p* < 0.001), muscle fiber minimum Feret’s diameter (W = 59, *p* < 0.01), L* (W = 271.5, *p* < 0.05) and hue angle (W = 274, *p* < 0.05). The medians of the above traits were lower for females than males; the only exception was live weight. Furthermore, larger interquartile ranges were observed for females, except for muscle fiber minimum Feret’s diameter (Table 3). Results of Kruskal–Wallis one-way analyses of variance show statistically significant differences between different LWCs of fat-tailed sheep (Table 4) regarding all body (live weight: H(4) = 138.39, wither height: H(4) = 120.37—*p* < 0.001) and most carcass (carcass length: H(4) = 118.27, hot carcass weight: H(4) = 132.36, carcass yield: H(4) = 85.46—*p* < 0.001) and meat quality traits (L*: H(4) = 24.526—*p* < 0.001, hue angle: H(4) = 10.646—*p* < 0.05, meat pH: H(4) = 12.333—*p* < 0.05, hardness 1: H(4) = 14.017—*p* < 0.01, hardness 2: H(4) = 13.978—*p* < 0.01, springiness: H(4) = 11.405—*p* < 0.05, chewiness: H(4) = 16.796—*p* < 0.01). Statistically significant (*p* < 0.05) pairwise comparisons between mean ranks of LWC for each of the above traits are presented in Figure 1. Regarding body and carcass traits, almost all differences between LWC were significant; the exceptions were 50% with 70% and 70% with 100% LWC. Moreover, wither height, carcass length and weight of 35% were not significantly different from that of 50% LWC, nor were carcass yields of 25% and 35% LWC. Few significant pairwise comparisons were observed regarding meat color and tenderness traits, most of which were observed for L* and meat chewiness; L* of 25% and 35% LWC differed from that of 70% and 100%, whereas meat chewiness of 25% was different from 35% and 50% LWC and that of 50% from 100% LWC. Differences regarding physicochemical characteristics of meat were not statistically significant (*p* > 0.05).

### 3.2. Comparison of Fat-Tailed and Thin-Tailed Sheep Carcasses

#### 3.2.1. The Effect of Sheep Population

Mann Whitney U tests revealed statistically significant differences between fat-tailed and thin-tailed sheep regarding carcass yield (W = 8544.5, *p* < 0.01) and a* (W = 923.5, *p* < 0.05). The median carcass yield of fat-tailed sheep was 53.74% compared to 48.77% for thin-tailed sheep, whereas for a*, the respective values were 12.44 and 13.13 (Table 5).

#### 3.2.2. Effects of the Interaction of Sheep Population with LWC 

Statistically significant results of the two-way ANOVA, including the effects of the interactions of sheep population with LWC on the studied traits, are presented in Table 6. A detailed version of the table also containing non-significant results can be found in the supplementary materials section (Table S1a). In the aforementioned analyses, the effect of sheep population was statistically significant for wither height (f(1) = 5.53, *p* < 0.05); compared to fat-tailed sheep, thin-tailed sheep were on average shorter by 0.01 m. The respective significant effects of the Scheirer–Ray–Hare tests are presented in Table 7 (detailed version of the table provided in the supplementary materials section—Table S2a); results show that sheep population significantly affected carcass yield (H(1) = 7.44, *p* < 0.01) and a* (H(1) = 4.23, *p* < 0.05). 

**Figure 2:**
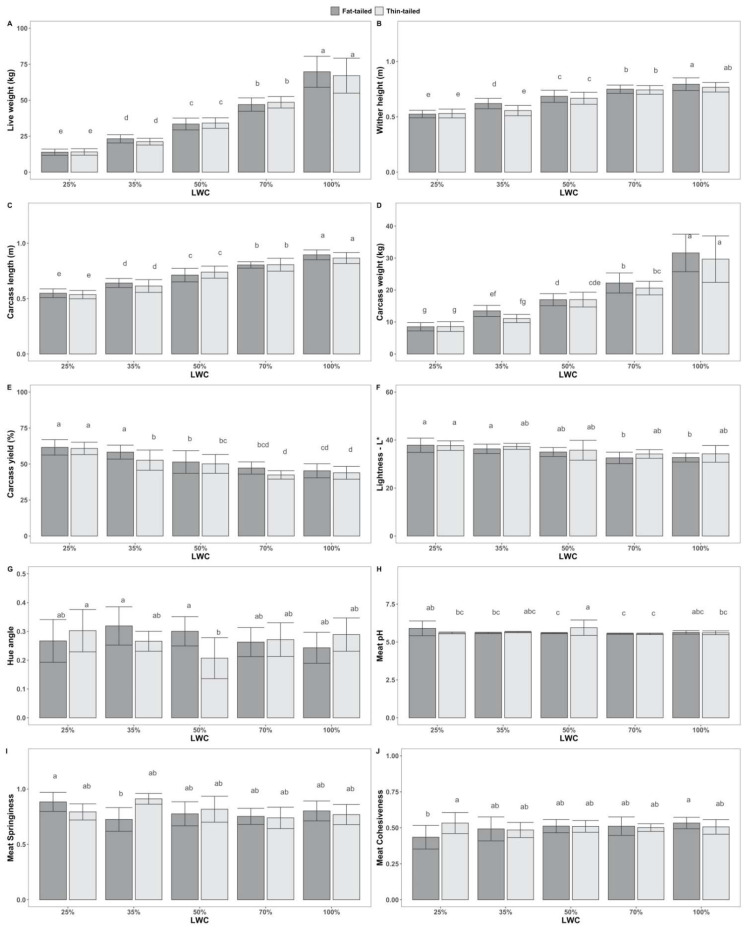
Tukey’s post hoc tests for pairwise comparisons between groups, following two-way ANOVA, accounting for the effect of the interaction between live-weight categories (LWC—25, 35, 50, 70 and 100% of mature live weight) and sheep populations (fat-tailed, thin-tailed) on the tested traits: (**A**) Live weight—in kg; (**B**) Wither height—in m; (**C**) Carcass length—in m; (**D**) Carcass weight—in kg; (**E**) Carcass yield—as %; (**F**) Lightness—L*; (**G**) Hue angle; (**H**) Meat pH; (**I**) Meat Springiness; (**J**) Meat Cohesiveness. Superscripts not sharing any common letter between groups indicate statistically significant differences (*p* < 0.05).

ANOVA results indicate also that the interaction of sheep population with LWC had significant effects on wither height (f(4) = 3.45, *p* < 0.01), b* (f(4) = 2.56, *p* < 0.05), hue angle (f(4) = 4.63, *p* < 0.01) and springiness (f(4) = 3.77, *p* < 0.01). Tukey’s post hoc tests revealed statistically significant pairwise differences between groups, as shown in Figure 2. Scheirer–Ray–Hare tests showed that meat cohesiveness (H(4) = 11.84, *p* < 0.05) and carcass pH (H(4) = 9.80, *p* < 0.05) were significantly affected by the interaction of sheep population with LWC. 

#### 3.2.3. Effects of the Interaction of Sheep Population with Sex

When accounting for the effects of the interactions of sheep population with sex, ANOVA results (significant effects presented in Table 6—full version including all effects in Table S1b) show that sheep population had no significant effect on the studied traits (*p* > 0.05). Scheirer–Ray–Hare tests (Table 7 and Table S2b present significant effects only and total effects, respectively) showed that thin-tailed sheep had significantly lower carcass yield (H(1) = 7.44, *p* < 0.01). 

**Figure 3:**
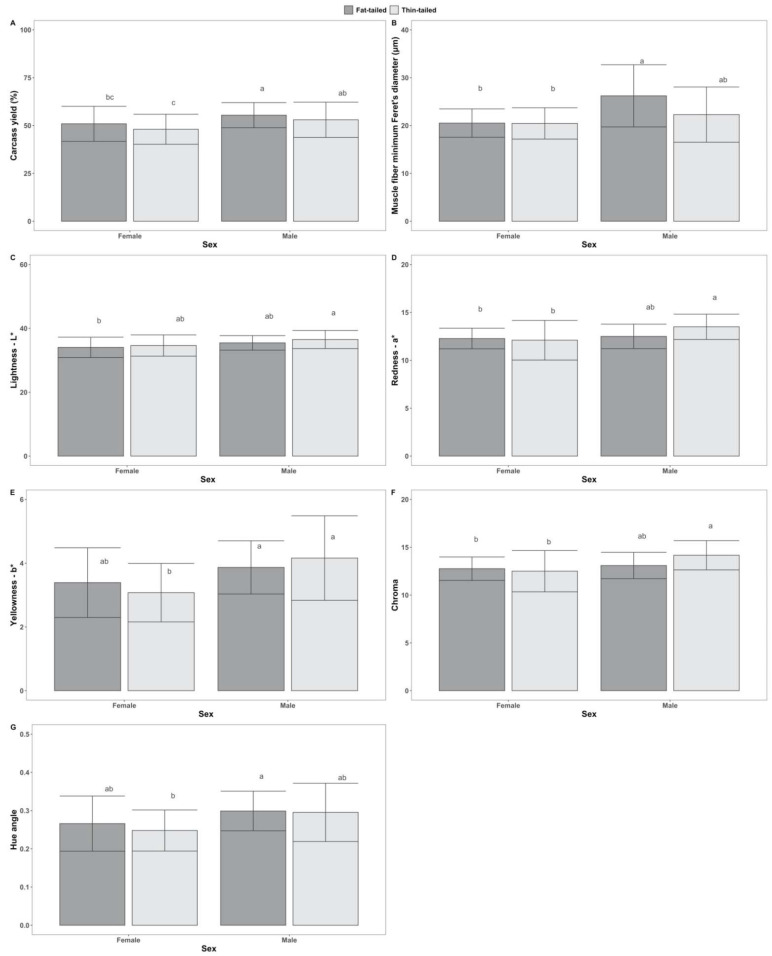
Tukey’s post hoc tests for pairwise comparisons between groups, following two-way ANOVA, accounting for the effect of the interaction between sex (male, female) and sheep populations (fat-tailed, thin-tailed) on the tested traits: (**A**) Carcass yield—as %; (**B**) Muscle fiber minimum Feret’s diameter—in μm; (**C**) Lightness—L*; (**D**) Redness—a*; (**E**) Yellowness—b*; (**F**) Chroma; (**G**) Hue angle. Superscripts not sharing any common letter between groups indicate statistically significant differences (*p* < 0.05).

Regarding the interaction of sheep population with sex, significant effects were found on a* (f(1) = 4.03, *p* < 0.05) and chroma (f(1) = 4.37, *p* < 0.05), based on ANOVA. Results of Tukey’s post hoc tests that were statistically significant are presented in Figure 3. Moreover, a significant effect of the interaction was found on carcass pH (H(1) = 4.67, *p* < 0.05) according to Scheirer–Ray–Hare tests.

## 4. Discussion

### 4.1. Quality Assessment of Fat-Tailed Sheep Carcasses

Mean values of body and carcass traits of fat-tailed sheep reported herein present both similarities and differences compared to respective estimations of relevant studies on Chios and other fat-tailed sheep breeds. Wither height was similar to that reported in literature (ca. 0.60 m) for Chios crosses of live weight corresponding to 35% LWC of the present study [21,22]. Present results of mean wither height for 70 and 100% LWC are also in general accordance with those of Awassi (Jordan, Turkey), Red Karaman (Turkey), Barki (Libya, Egypt), Ossimi and Rahmani (Egypt); averages ranged from 0.72 to 0.74 m [23,24,25]. However, adult sheep of other fat-tailed breeds reared in the Mediterranean basin, Middle East and Northern Africa had lower mean wither height (0.54–0.70 m) [26].

Regarding carcass traits, Obeidat et al. [21] reported lower mean hot carcass weight (9.2 kg) and yield (47.0%) of Chios crosses corresponding to 35% LWC, whereas Ekiz et al. [27] presented lower mean carcass yield (54.6%) but slightly higher mean carcass weight (14.6 kg) and length (0.69 m). Chios crosses with a mean live weight corresponding to 50% LWC presented lower mean carcass weights (12.5–13.2 kg, depending on management practices) and yields (44.2–45.6%) compared to present results [28]. Differences regarding carcass dressing between the present study and the previous studies may be the underlying cause of the observed deviations. Herein, light and midweight carcasses (25%, 35% and 50% LWC) included head and some viscera, resulting in higher yields, whereas, in the other studies, these carcass parts were removed during dressing. Implementation of a uniform carcass dressing across all studies would possibly limit the observed discrepancies. Earlier studies [29,30], concerning Chios lambs slaughtered at approximately 50% of mature live weight and dressed as herein, reported similar (16.5 kg) and slightly higher mean carcass weights (20.3 kg) and yields (54.6% and 59.5%, respectively) compared to ours. Although these studies are about sheep of the same breed and similar live weight as the present ones, they are quite dated; hence, it is likely that the studied population has evolved, causing the observed discrepancies. Soycan Önenç et al. [31] have found that intensively reared Chios sheep of live weight corresponding to 70% LWC produced carcasses marginally lighter (20.4 kg) and shorter in length (0.61 m) that yielded less (40.8%). The observed differences with the present study most likely reflect true variability between populations and management practices, given that mean live weights and carcass dressing were similar. 

Concerning carcasses of other fat-tailed sheep breeds, highly variable length averages (0.53–1.04 m for adult sheep and 0.44–0.99 m for lambs) have been reported [26]; such results are not directly comparable with ours, since values of studied traits were averaged based on the age of the animals, whereas in the present study, data were distinguished based on sheep live weight or sex. Studies reviewed by Mohapatra and Shinde [32] reported that Awassi, Karaman and indigenous Iranian fat-tailed sheep in 50 and 70% LWC presented high variability regarding mean carcass weights (12.2–18.9 kg and 19.1–26.4 kg, respectively) and yields (39.7–49.7% and 46.0–50.8%, respectively). In the present study, respective values of 70% LWC are within the above ranges, whereas mean yield of 50% LWC is slightly higher, probably due to carcass dressing differences discussed above. Awassi carcasses corresponding to the present study’s 35, 50 and 70% LWC had lower mean weights (8.5, 14.1 and 18.4, respectively) and higher yield (ca. 50%), except for the 35% LWC, as expected due to the dissimilar dressing [33]. Fat-tailed Iranian Chaal and Zandi and Egyptian Ossimi, Barki and Rahmani sheep, fattened up to live weights within 70% LWC, presented higher mean carcass yield (53.5–58.2% for Iranian and 53.1–56.2% for Egyptian breeds) and ranging mean carcass weight (slightly lower for Iranian, 20.1–21.5 kg, and higher for Egyptian, 24.3–26.1 kg) [25,34]. Since carcass dressing was similar to that of the present study, the higher yield of Iranian and Egyptian fat-tailed sheep carcasses can be attributed at least partly to the fattening periods that preceded slaughter; it is likely that fattening of Greek fat-tailed Chios sheep and their crosses may also increase carcass yields. 

Studies regarding carcass pH and meat quality parameters (pH/color/tenderness) of Chios sheep are rather scarce. In agreement with the present results, Ekiz et al. [27] reported a mean carcass pH of ca. 6.5, measured approximately 45 min after slaughter, for Chios sheep with mean live weight corresponding to 35% LWC. They also report slightly higher L* and a* and ranging b* values. Relevant studies have reported higher L*, b* and chroma values of meat samples from Awassi, indigenous Turkish and Iranian fat-tailed sheep corresponding to 35%, 50% and 70% LWC of the present study [35,36,37]. Higher L* and a* and lower b* values were reported for Kangal Akkaraman sheep [38]. Slightly higher values of all color parameters were reported for meat of Awassi sheep in 70% LWC [39]. In all the above cases, measurements were performed in a smaller time interval after slaughter (24 h) and different sample preparation, illuminant, aperture size and observer angle were implemented compared to the present study. Such differences may partly explain the observed discrepancies. This hypothesis is further supported by the fact that in our study, meat color parameters changed among LWCs following trends similar to other studies, in which heavier carcasses produced darker meat (higher L*), whereas a* and b* were approximately the same regardless of carcass weight [36]. Thus, color parameter differences of other studies and the present one may reflect different methods and equipment settings used. In most of the above studies, meat tenderness was assessed, as well; Warner–Bratzler shear force tests and cooked meat were used, and hence results are not comparable to the present ones. 

Muscle fiber minimum Feret’s diameter, a trait commonly used in human and veterinary medical studies involving investigation of muscle fiber microstructure, is rarely used for meat quality assessment. However, increased muscle fibers’ diameter is important to the meat production industry, since it has been correlated with higher meat yield and more tender meat [40]. Relevant studies on pigs, beef and avian species have been published recently [40,41,42,43,44,45]; to our knowledge, no relevant studies on sheep are available. Results of the present study show a tendency for increased diameter of muscle fibers in heavier carcasses. Fat-tailed sheep in 70% LWC presented the largest diameter. The latter suggests that young adult sheep that have not reached the end of their dairy productive life are more likely to produce carcasses of higher quality; normally, sheep at this productive stage have not been exposed to stress and challenges related to consecutive reproductive periods and lactations, and hence they retain a healthier, balanced and robust body conformation. 

Concerning the physicochemical characteristics of meat, slightly higher protein and lipid contents and lower moisture were observed in our study compared to other relevant ones [36,37,39]; light carcasses (corresponding to 35% LWC) were the exception, in which case present results indicated lower protein content. In all cases, differences regarding protein content were slight (ca. 1–2%), whereas lipid contents differed more strongly (ranging roughly from 2 to 8%). The lower moisture content is most likely caused by the thawing process to which the present samples were subjected; in the other studies, fresh samples were used. Given the lower moisture content of our samples, the higher protein and fat contents may be attributed at least partly to condensing. Nevertheless, fat content differences are relatively large, possibly indicating an actual trend of higher fat content in Chios sheep carcasses compared to other breeds. 

In agreement with studies on Chios or other fat-tailed sheep [46,47], statistically significant differences between male and female sheep were detected, regarding live weight, carcass yield, muscle fiber minimum Feret’s diameter, L* and hue angle. Interestingly, females presented higher live weight; this could be associated with the fact that carcasses of female sheep in the heavier LWC slightly outnumbered those of male sheep. On the contrary, male carcass yield and muscle fiber minimum Feret’s diameter were larger. This agrees with the fact that sex-related anatomical differences generally render male sheep more muscular than female. Color parameter differences suggest that meat of female sheep is darker (lower L*) and more reddish (lower hue angle); however, they are very slight and need to be interpreted with caution. Likewise, Yousefi et al. [36] reported that female Chaal lambs had statistically significant higher a*, which also results in more reddish meat. 

Overall, in previous studies, largely ranging values were observed for most studied traits. Categorization of sheep and their carcasses according to their weight facilitated comparisons between the present study and other studies. The importance of this categorization is underpinned by the fact that the observed differences among LWCs of the present study were statistically significant for most traits. Considering the absence of quality-improving feed interventions prior to slaughter and given that all studied animals are considered “by-products” of dairy production, slaughtered at different live weights and productive stages, large variability was expected regarding the studied traits. However, this indicates that there may be space for improving fat-tailed sheep carcass and meat quality. The different LWCs defined in the present study equally represent very light lamb carcasses (25% LWC) and heavy carcasses of adult sheep (100% LWC), which are mainly produced in Greece, as well as midweight carcasses, which are generally scarce (35, 50, 70% LWC). Comparison with the respective data of other mentioned studies implies improvement potential through implementing appropriate fattening protocols.

Considering all tested traits, fat-tailed sheep in 50 and 70% LWC produce carcasses of the highest yield and best quality across all LWC. Specifically, they are characterized by muscle fibers with larger diameter, lower L* and hue angle, higher a* and chroma, moderate b*, lower values of TPA parameters, higher moisture and protein content and lower lipid content. For all the above traits, except for chroma, a*, b*, meat cohesiveness and meat composition traits, the effect of LWC as well as some differences between groups were statistically significant (*p* < 0.05). Effects that did not reach statistical significance showed a positivity tendency, implying higher quality of these carcasses; hence, further investigation on a larger sample is warranted. Carcasses of 50 and 70% LWC had higher moisture content, thicker muscle fibers, lower values of TPA parameters and meat composition that complied with consumer demands for leaner [4] and more tender meat [40,48]. Meat color profile indicated that meat was more reddish and less yellowish, with a more intense and vivid hue, which are relevant to the ideal bright red color that consumers associate with freshness [13,49]. Therefore, implementation of appropriate fattening protocols up to finishing weights corresponding to 50 and 70% LWC may increase meat yields (compared to currently common light carcasses of ca. 9 kg), improve meat quality and capitalization of fat-tailed sheep carcasses and overall boost the sheep meat industry. This is further supported by other studies reporting optimum finishing weights of 44 and 30 kg (corresponding to 70% and 50% LWC) for fat-tailed Kangal Akkaraman and Awassi sheep, respectively, that maximize meat quantity without compromising quality [33,50].

### 4.2. Comparison of Fat-Tailed and Thin-Tailed Sheep Carcasses

To support the validity of our inferences, two different statistical approaches were involved in the comparison of fat-tailed and thin-tailed sheep: Mann–Whitney U tests and either two-way ANOVA or its non-parametric equivalent Scheirer–Ray–Hare tests, depending on compliance of the distribution of each studied trait with the assumptions of ANOVA. Results of Mann–Whitney U tests were further assessed with two-way ANOVA that accounted also for the effects of LWC or sex and their interaction with sheep population; the latter explained part of the observed variation, otherwise attributed only to the sheep population effect. In cases where ANOVA assumptions were violated, Scheirer–Ray–Hare tests were performed to avoid misleading results; as non-parametric, the latter tests were considered more reliable for the interpretation of effects in such cases.

Results from the above analyses suggest that fat-tailed sheep carcass yield was higher than that of thin-tailed. This is in accordance with the study of Panopoulou et al. [29], in which the carcass yield of Chios sheep was higher (by 2.2%) compared to a thin-tailed indigenous Greek sheep breed, Karagouniko. On the contrary, Ekiz et al. [27] have found that Chios reared in Turkey yielded less than Merino and Kivircik thin-tailed sheep (by 2.6%). Regarding other fat-tailed breeds, Akkaraman sheep presented consistently higher carcass yield compared to thin-tailed Anatolian Merino under different feeding systems [51]. Furthermore, Iranian fat-tailed Chaal and Zandi sheep presented higher carcass yield than thin-tailed Zel sheep or their crosses; however, these differences were not always statistically significant [34,52]. Based on the above, fat-tailed sheep are not consistently presenting higher yields compared to thin-tailed. This could potentially be attributed to underlying differences among studied populations regarding management practices, sampling methods or sample size variance. However, the present results, supported by the evidence of another study concerning Greek sheep [29], suggest that Chios and their crosses achieve higher carcass yields compared to local thin-tailed populations.

Meat of the studied fat-tailed sheep had statistically significant lower a* compared to that of thin-tailed, based on statistical analyses performed. These findings are in accordance with other studies that have also reported respective differences between Chios and thin-tailed Kivircik and Imroz sheep [27] and between fat-tailed Chaal and thin-tailed Zel sheep [52]. Lower a* represents meat color that is more brownish than reddish, hence less desirable to consumers. However, in order to make safe assumptions, all color parameters should be considered. Present results indicate that chroma, b* and hue angle were affected by the interaction of sheep population with sex or LWC (*p* < 0.05). This observation is in accordance with Yousefi et al. [52], who have reported significantly different b* between fat-tailed and thin-tailed sheep. Overall, in the present study, sheep population affects meat color; fat-tailed sheep present a trend towards less desirable meat color; however, differences from thin-tailed are minimal. Further investigation of a larger sample size may facilitate quantifying the overall impact.

Wither height was significantly affected by sheep population and its interaction with LWC, emphasizing unique morphological features between the two populations; fat-tailed sheep were taller than thin-tailed by approximately 1 cm. However, due to the scarcity of similar studies concerning this trait, assessing the relevance of our observations is rather difficult. 

Statistically significant effects of the interaction of sheep population and LWC on meat springiness and cohesiveness were also detected, suggesting that meat of fat-tailed sheep is more tender (lower springiness and cohesiveness) compared to that of thin-tailed. Yousefi et al. [52] reported the opposite regarding Iranian thin-tailed Zel and fat-tailed Chaal lambs; however, assessment methods were different than those of the present study. Unique features of each population may be the underlying cause for the observed discrepancies.

The interactions with LWC and sex had statistically significant effects on carcass pH. Similar significant differences (*p* < 0.05) between carcasses of animals slaughtered at different live weights have been reported in literature for both fat-tailed and thin-tailed sheep, however, in these cases pH was measured at a larger time interval of 24 h; differences between sexes were not statistically significant [36,53]. 

Meat physicochemical characteristics did not differ between sheep populations. On the contrary, Aksoy et al. [37] have reported such differences between fat-tailed and thin-tailed sheep for one or more of the studied traits. In our study, the small sample size used for the relevant analyses may be insufficient for the detection of possible significant differences between groups or overall effects. In future studies, assessment of the above effects with a larger sample size may facilitate safe assumptions. Furthermore, evaluation of the fatty acid profile, which is commonly performed in relevant studies [36,37,52,54], may provide further insight.

## 5. Conclusions

The present results indicate that carcasses and meat of Chios fat-tailed sheep (and their crosses) reared in Greece present desirable characteristics, in most cases exceeding the performance of local thin-tailed sheep, which are generally considered to produce leaner meat of higher quality. Most of the studied traits were significantly affected by LWC and/or sex. Live weight at slaughter is key to maximizing meat production, capitalizing and minimizing waste. Present results suggest that fattening of lambs until finishing weights corresponding to 50 and 70% LWC could improve the otherwise low marketability of fat-tailed sheep carcasses, therefore contributing to the overall production efficiency and sustainability of the sector. 

## Figures and Tables

**Table 1:** Weight range per live-weight category (LWC).

	Live-Weight Categories (% of Mature Body Weight)				
	25%	35%	50%	70%	100%
Weight Range (kg)	<17.5	17.6–27.5	27.6–39.5	39.6–55	>55.1

**Table 2:** (**a**)**.** Descriptive statistics for body, carcass and meat quality traits of fat-tailed and thin-tailed sheep per live-weight category (LWC). (**b**) Descriptive statistics for body, carcass and meat quality traits of fat-tailed and thin-tailed sheep per sex.

**(a)**											
		**Live-Weight Category (% of Mature Body Weight)**									
		**25%**		**35%**		**50%**		**70%**		**100%**	
**Trait**	**Sheep**	***n***	**Mean (SD)**	***n***	**Mean (SD)**	***n***	**Mean (SD)**	***n***	**Mean (SD)**	***n***	**Mean (SD)**
Live weight (kg)	Fat-tailed	27	13.90 (2.176)	38	23.20 (2.874)	25	33.46 (4.106)	24	46.96 (4.646)	32	69.77 (10.758)
	Thin-tailed	25	14.07 (2.241)	13	21.22 (2.365)	14	34.12 (3.619)	18	48.57 (3.973)	27	67.08 (12.135)
Wither height (m)	Fat-tailed	27	0.52 (0.034)	38	0.62 (0.047)	25	0.69 (0.055)	24	0.75 (0.037)	32	0.79 (0.057)
	Thin-tailed	25	0.53 (0.040)	13	0.56 (0.046)	14	0.67 (0.055)	18	0.74 (0.039)	27	0.77 (0.043)
Carcass length (m)	Fat-tailed	25	0.55 (0.039)	38	0.64 (0.041)	25	0.71 (0.061)	19	0.80 (0.029)	30	0.90 (0.045)
	Thin-tailed	25	0.54 (0.038)	13	0.61 (0.057)	13	0.74 (0.055)	12	0.81 (0.058)	21	0.87 (0.050)
Hot carcass weight (kg)	Fat-tailed	27	8.52 (1.289)	38	13.48 (1.731)	25	16.98 (1.891)	24	22.20 (3.133)	32	31.59 (5.863)
	Thin-tailed	25	8.57 (1.523)	13	11.09 (1.298)	14	17.00 (2.315)	18	20.60 (2.130)	27	29.66 (7.263)
Carcass yield (%)	Fat-tailed	27	61.59 (5.369)	38	58.27 (4.836)	25	51.41 (7.854)	24	47.23 (4.231)	32	45.28 (4.878)
	Thin-tailed	25	60.84 (4.294)	13	52.67 (7.014)	14	50.09 (6.533)	18	42.42 (2.907)	27	43.92 (4.460)
Muscle fiber minimum Feret’s diameter (μm)	Fat-tailed	1	NA	2	19.57 (0.515)	3	19.28 (1.445)	12	23.91 (4.484)	16	22.81 (6.172)
	Thin-tailed	8	17.34 (1.960)	5	20.52 (3.996)	7	22.75 (2.891)	5	20.55 (2.463)	17	22.97 (5.657)
Carcass pH	Fat-tailed	25	6.31 (0.208)	37	6.49 (0.206)	23	6.36 (0.246)	22	6.43 (0.286)	32	6.43 (0.345)
	Thin-tailed	25	6.35 (0.230)	13	6.26 (0.216)	14	6.27 (0.277)	16	6.46 (0.324)	26	6.48 (0.254)
Lightness—L*	Fat-tailed	7	37.84 (2.947)	15	36.30 (1.966)	15	35.00 (1.901)	11	32.55 (2.393)	10	32.69 (1.865)
	Thin-tailed	10	37.66 (1.996)	3	37.35 (1.257)	8	35.75 (4.141)	8	34.19 (1.784)	13	34.25 (3.505)
Redness—a*	Fat-tailed	7	11.65 (1.464)	15	12.41 (0.727)	15	12.66 (0.678)	11	12.50 (1.219)	10	12.37 (1.945)
	Thin-tailed	10	12.96 (1.234)	3	12.35 (1.193)	8	13.72 (1.541)	8	12.79 (1.200)	13	12.22 (2.666)
Yellowness—b*	Fat-tailed	7	3.17 (0.962)	15	4.11 (0.907)	15	3.94 (0.788)	11	3.40 (0.885)	10	3.15 (1.142)
	Thin-tailed	10	4.10 (1.291)	3	3.39 (0.724)	8	2.96 (1.314)	8	3.62 (0.988)	13	3.71 (1.394)
Chroma	Fat-tailed	7	12.10 (1.484)	15	13.10 (0.789)	15	13.27 (0.791)	11	12.97 (1.359)	10	12.79 (2.145)
	Thin-tailed	10	13.63 (1.469)	3	12.81 (1.328)	8	14.07 (1.747)	8	13.31 (1.368)	13	12.79 (2.910)
Hue angle	Fat-tailed	7	0.27 (0.074)	15	0.32 (0.066)	15	0.30 (0.051)	11	0.26 (0.051)	10	0.24 (0.054)
	Thin-tailed	10	0.30 (0.074)	3	0.27 (0.035)	8	0.21 (0.071)	8	0.27 (0.059)	13	0.29 (0.058)
Meat pH	Fat-tailed	7	5.90 (0.487)	15	5.60 (0.046)	15	5.59 (0.038)	11	5.55 (0.041)	10	5.64 (0.118)
	Thin-tailed	10	5.60 (0.062)	3	5.66 (0.044)	8	5.94 (0.503)	8	5.54 (0.049)	13	5.61 (0.128)
Meat hardness 1 (g)	Fat-tailed	7	1726.34 (770.736)	15	1058.61 (970.328)	15	706.80 (266.121)	11	861.07 (502.399)	10	1328.21 (660.300)
	Thin-tailed	10	1117.26 (981.417)	3	1248.02 (483.990)	8	1255.87 (533.473)	8	1219.58 (715.994)	12	1447.93 (933.199)
Meat hardness 2 (g)	Fat-tailed	7	1306.80 (584.397)	15	822.40 (664.038)	15	579.15 (219.401)	11	687.85 (387.361)	10	1076.70 (521.397)
	Thin-tailed	10	899.91 (759.907)	3	1000.27 (383.441)	8	1020.33 (426.026)	8	967.40 (547.251)	12	1137.00 (709.939)
Meat springiness	Fat-tailed	7	0.88 (0.087)	15	0.73 (0.107)	15	0.78 (0.109)	11	0.75 (0.073)	10	0.80 (0.090)
	Thin-tailed	10	0.79 (0.073)	3	0.91 (0.049)	8	0.82 (0.117)	8	0.74 (0.097)	12	0.77 (0.091)
Meat cohesiveness	Fat-tailed	7	0.43 (0.082)	15	0.49 (0.083)	15	0.51 (0.046)	11	0.51 (0.064)	10	0.53 (0.040)
	Thin-tailed	10	0.53 (0.073)	3	0.48 (0.053)	8	0.51 (0.041)	8	0.50 (0.027)	12	0.51 (0.051)
Meat chewiness	Fat-tailed	7	646.14 (298.243)	15	351.05 (263.031)	15	277.23 (103.884)	11	329.67 (192.222)	10	571.88 (312.895)
	Thin-tailed	10	450.53 (366.955)	3	549.16 (225.129)	8	524.34 (224.746)	8	462.32 (291.320)	12	557.09 (343.354)
Meat moisture (%)	Fat-tailed	7	62.94 (3.312)	4	61.16 (4.825)	5	60.21 (4.281)	5	62.93 (10.044)	4	66.14 (4.893)
	Thin-tailed	7	63.48 (6.743)	1	NA	3	61.92 (3.957)	2	45.61 (20.365)	6	61.53 (7.719)
Meat lipid content (%)	Fat-tailed	7	5.86 (2.416)	4	9.11 (6.721)	5	11.09 (5.856)	5	6.80 (5.764)	4	5.04 (2.608)
	Thin-tailed	7	5.44 (5.860)	0	NA	3	3.13 (0.436)	2	11.59 (13.774)	6	8.75 (6.756)
Meat protein content (%)	Fat-tailed	7	19.69 (5.181)	4	16.64 (5.149)	5	22.79 (3.108)	5	22.35 (3.288)	4	23.40 (2.215)
	Thin-tailed	7	22.81 (1.186)	0	NA	3	18.71 (0.385)	2	17.38 (3.811)	6	23.71 (2.810)
**(b)**											
						**Sex**					
						**Female**		**Male**		**Total**	
**Trait**				**Sheep**		***n***	**Mean (SD)**	***n***	**Mean (SD)**	***n***	**Mean (SD)**
Live weight (kg)				Fat-tailed		77	40.34 (20.245)	69	34.02 (21.351)	146	37.35 (20.943)
				Thin-tailed		58	42.42 (22.011)	39	34.11 (21.848)	97	39.08 (22.213)
Wither height (m)				Fat-tailed		77	0.69 (0.100)	69	0.66 (0.112)	146	0.67 (0.106)
				Thin-tailed		58	0.68 (0.106)	39	0.64 (0.114)	97	0.66 (0.111)
Carcass length (m)				Fat-tailed		70	0.73 (0.138)	67	0.70 (0.118)	137	0.72 (0.130)
				Thin-tailed		45	0.72 (0.147)	39	0.68 (0.138)	84	0.70 (0.143)
Hot carcass weight (kg)				Fat-tailed		77	19.22 (8.296)	69	17.84 (9.197)	146	18.56 (8.730)
				Thin-tailed		58	19.38 (9.696)	39	16.52 (8.414)	97	18.23 (9.264)
Carcass yield (%)				Fat-tailed		77	50.91 (9.164)	69	55.44 (6.566)	146	53.05 (8.330)
				Thin-tailed		58	48.08 (7.854)	39	53.02 (9.216)	97	50.07 (8.727)
Muscle fiber minimum Feret’s diameter (μm)				Fat-tailed		22	20.52 (2.959)	12	26.22 (6.499)	34	22.53 (5.224)
				Thin-tailed		23	20.45 (3.269)	19	22.29 (5.777)	42	21.28 (4.609)
Carcass pH				Fat-tailed		74	6.39 (0.276)	65	6.43 (0.258)	139	6.41 (0.268)
				Thin-tailed		57	6.42 (0.267)	37	6.31 (0.261)	94	6.38 (0.269)
Lightness—L*				Fat-tailed		26	34.04 (3.197)	32	35.45 (2.276)	58	34.82 (2.793)
				Thin-tailed		21	34.62 (3.328)	21	36.49 (2.847)	42	35.56 (3.202)
Redness—a*				Fat-tailed		26	12.27 (1.076)	32	12.49 (1.283)	58	12.39 (1.190)
				Thin-tailed		21	12.10 (2.062)	21	13.50 (1.325)	42	12.80 (1.852)
Yellowness—b*				Fat-tailed		26	3.39 (1.092)	32	3.87 (0.834)	58	3.65 (0.979)
				Thin-tailed		21	3.08 (0.917)	21	4.16 (1.325)	42	3.62 (1.253)
Chroma				Fat-tailed		26	12.76 (1.226)	32	13.09 (1.375)	58	12.95 (1.310)
				Thin-tailed		21	12.50 (2.159)	21	14.16 (1.529)	42	13.33 (2.030)
Hue angle				Fat-tailed		26	0.27 (0.072)	32	0.30 (0.052)	58	0.28 (0.063)
				Thin-tailed		21	0.25 (0.054)	21	0.30 (0.076)	42	0.27 (0.069)
Meat pH				Fat-tailed		26	5.66 (0.272)	32	5.60 (0.103)	58	5.63 (0.198)
				Thin-tailed		21	5.74 (0.354)	21	5.58 (0.066)	42	5.66 (0.264)
Meat hardness 1 (g)				Fat-tailed		26	1083.37 (728.458)	32	1036.00 (740.972)	58	1057.23 (729.320)
				Thin-tailed		20	1242.28 (860.706)	21	1297.61 (735.765)	41	1270.62 (789.523)
Meat hardness 2 (g)				Fat-tailed		26	868.72 (568.620)	32	809.92 (510.389)	58	836.28 (533.249)
				Thin-tailed		20	983.81 (656.025)	21	1041.41 (571.015)	41	1013.31 (606.881)
Meat springiness				Fat-tailed		26	0.78 (0.096)	32	0.77 (0.113)	58	0.78 (0.105)
				Thin-tailed		20	0.79 (0.101)	21	0.79 (0.096)	41	0.79 (0.098)
Meat cohesiveness				Fat-tailed		26	0.51 (0.057)	32	0.49 (0.076)	58	0.50 (0.068)
				Thin-tailed		20	0.50 (0.054)	21	0.52 (0.048)	41	0.51 (0.052)
Meat chewiness				Fat-tailed		26	428.51 (304.327)	32	379.73 (226.493)	58	401.59 (262.905)
				Thin-tailed		20	467.90 (308.323)	21	541.58 (298.459)	41	505.64 (301.801)
Meat moisture (%)				Fat-tailed		11	60.68 (6.833)	14	64.14 (4.227)	25	62.62 (5.674)
				Thin-tailed		10	59.65 (11.998)	8	61.75 (5.600)	18	60.59 (9.501)
Meat lipid content (%)				Fat-tailed		11	8.82 (5.802)	14	6.43 (4.015)	25	7.48 (4.921)
				Thin-tailed		10	8.16 (7.441)	8	5.20 (5.470)	18	6.84 (6.627)
Meat protein content (%)				Fat-tailed		11	21.59 (3.018)	14	20.44 (5.364)	25	20.95 (4.441)
				Thin-tailed		10	22.28 (3.764)	8	21.25 (1.979)	18	21.82 (3.064)

NA: Not applicable.

**Table 3:** Results of non-parametric Mann–Whitney U tests performed to compare between sexes of fat-tailed sheep (rejection of null hypothesis when *p* < 0.05: Differences between medians of groups are statistically significant, hence groups come from different populations).

	Female			Male			
Traits	*n*	Median	Interquartile Range	*n*	Median	Interquartile Range	*p*-Value
Live weight (kg)	77	39.00	32.00	69	26.50	23.40	<0.05
Wither height (m)	77	0.71	0.13	69	0.65	0.20	0.088
Carcass length (m)	70	0.77	0.21	67	0.66	0.18	0.132
Hot carcass weight (kg)	77	17.80	12.00	69	15.00	9.20	0.143
Carcass yield (%)	77	48.71	13.45	69	56.60	9.02	<0.001
Muscle fiber minimum Feret’s diameter (μm)	22	19.83	3.68	12	23.98	11.01	<0.01
Carcass pH	74	6.36	0.45	65	6.45	0.33	0.329
Lightness—L*	26	33.75	5.60	32	34.97	2.92	<0.05
Redness—a*	26	12.46	1.50	32	12.37	1.19	0.633
Yellowness—b*	26	3.16	1.74	32	4.01	1.03	0.081
Chroma	26	12.85	1.50	32	13.02	1.44	0.412
Hue angle	26	0.24	0.11	32	0.30	0.06	<0.05
Meat pH	26	5.60	0.08	32	5.59	0.09	0.256
Hardness 1 (g)	26	942.70	1106.05	32	874.54	570.02	0.907
Hardness 2 (g)	26	753.02	815.43	32	713.49	442.66	0.857
Springiness	26	0.79	0.14	32	0.77	0.17	0.790
Cohesiveness	26	0.51	0.07	32	0.51	0.08	0.595
Chewiness	26	357.28	391.83	32	350.22	227.88	0.809
Meat moisture (%)	11	60.50	5.38	14	64.08	4.51	0.106
Meat lipid content (%)	11	7.68	7.93	14	5.34	4.88	0.311
Meat protein content (%)	11	21.92	2.94	14	22.32	3.64	0.891

**Table 4:** Results of non-parametric Kruskal–Wallis one-way analyses of variance for assessing the statistical significance of the differences between mean ranks of the studied traits for different live-weight categories (LWC—% of mature live weight) of fat-tailed sheep (rejection of null hypothesis when *p* < 0.05: There are statistically significant differences between mean ranks of groups).

	LWC ^1^					
Traits	25%	35%	50%	70%	100%	*p*-Value
Live weight (kg)	14.000 ^a^	46.500 ^b^	78.000 ^c^	102.833 ^cd^	130.250 ^d^	<0.001
Wither height (m)	16.611 ^a^	48.855 ^b^	77.420 ^bc^	106.813 ^cd^	122.719 ^d^	<0.001
Carcass length (m)	15.820 ^a^	47.434 ^b^	70.560 ^bc^	97.184 ^cd^	121.483 ^d^	<0.001
Hot carcass weight (kg)	14.370 ^a^	48.303 ^b^	77.000 ^bc^	102.750 ^cd^	128.641 ^d^	<0.001
Carcass yield (%)	115.630 ^a^	101.605 ^a^	65.780 ^bc^	42.667 ^cd^	33.734 ^d^	<0.001
Carcass pH	52.080 ^a^	81.338 ^a^	62.609 ^a^	71.068 ^a^	75.469 ^a^	0.055
Lightness—L*	45.429 ^a^	39.200 ^a^	30.867 ^ab^	14.091 ^b^	18.700 ^b^	<0.001
Redness—a*	21.786 ^a^	29.767 ^a^	33.233 ^a^	30.500 ^a^	27.800 ^a^	0.674
Yellowness—b*	21.429 ^a^	37.100 ^a^	34.000 ^a^	25.500 ^a^	21.400 ^a^	0.071
Chroma	19.571 ^a^	32.000 ^a^	33.733 ^a^	29.545 ^a^	26.300 ^a^	0.399
Hue angle	26.714 ^ab^	37.533 ^a^	34.467 ^ab^	23.909 ^ab^	18.100 ^b^	<0.05
Meat pH	43.929 ^a^	31.933 ^ab^	28.533 ^ab^	16.364 ^b^	31.650 ^ab^	<0.05
Hardness 1 (g)	45.714 ^a^	27.367 ^ab^	20.867 ^b^	25.682 ^ab^	38.500 ^ab^	<0.01
Hardness 2 (g)	44.857 ^a^	27.400 ^ab^	21.000 ^b^	25.182 ^ab^	39.400 ^ab^	<0.01
Springiness	46.214 ^a^	21.033 ^b^	29.900 ^ab^	26.636 ^ab^	33.050 ^ab^	<0.05
Cohesiveness	13.500 ^a^	28.733 ^a^	30.767 ^a^	31.136 ^a^	38.150 ^a^	0.058
Chewiness	46.429 ^a^	24.400 ^bc^	21.600 ^bd^	26.000 ^acd^	41.000 ^ac^	<0.01
Meat moisture (%)	12.714 ^a^	11.000 ^a^	9.200 ^a^	15.600 ^a^	17.000 ^a^	0.486
Meat lipid content (%)	11.571 ^a^	14.750 ^a^	17.000 ^a^	11.800 ^a^	10.250 ^a^	0.617
Meat protein content (%)	10.714 ^a^	5.250 ^a^	16.400 ^a^	15.200 ^a^	17.750 ^a^	0.082

^1^ % of mature live weight; ^a,b,c,d^ Mean ranks not sharing any common letter are significantly different according to Dunn’s tests (*p* < 0.05).

**Table 5:** Results of non-parametric Mann–Whitney U tests performed to compare fat-tailed sheep to thin-tailed sheep (rejection of null hypothesis when *p* < 0.05: Differences between medians of groups are statistically significant, hence groups come from different populations).

	Fat-Tailed			Thin-Tailed			
Traits	*n*	Median	Interquartile Range	*n*	Median	Interquartile Range	*p*-Value
Live weight (kg)	77	30.50	30.38	58	37.00	38.50	0.751
Wither height (m)	77	0.68	0.16	58	0.68	0.20	0.535
Carcass length (m)	70	0.69	0.20	45	0.72	0.26	0.376
Hot carcass weight (kg)	77	16.60	10.95	58	17.00	13.20	0.691
Carcass yield (%)	77	53.74	0.13	58	48.77	0.14	<0.01
Muscle fiber minimum Feret’s diameter (μm)	22	21.26	5.22	23	20.37	6.12	0.318
Carcass pH	74	6.41	0.41	57	6.37	0.35	0.258
Lightness—L*	26	34.55	3.95	21	35.13	3.84	0.327
Redness—a*	26	12.44	1.49	21	13.13	1.85	<0.05
Yellowness—b*	26	3.65	1.57	21	3.54	1.61	0.618
Chroma	26	12.96	1.68	21	13.73	1.99	0.088
Hue angle	26	0.29	0.10	21	0.27	0.09	0.333
Meat pH	26	5.59	0.09	21	5.61	0.13	0.900
Hardness 1 (g)	26	934.19	754.52	20	1100.21	1061.88	0.141
Hardness 2 (g)	26	740.98	598.26	20	877.89	853.22	0.131
Springiness	26	0.78	0.16	20	0.78	0.11	0.511
Cohesiveness	26	0.51	0.08	20	0.51	0.07	0.741
Chewiness	26	350.22	267.45	20	453.24	471.23	0.100
Meat moisture (%)	11	63.06	5.27	10	63.54	8.43	0.815
Meat lipid content (%)	11	6.72	5.30	10	3.87	4.51	0.237
Meat protein content (%)	11	21.92	3.42	10	22.04	4.59	0.777

**Table 6:** (**a**) Statistically significant effects (*p* < 0.05) of sheep population (fat-tailed or thin-tailed sheep), live-weight category (LWC—% of mature live weight) and their interaction on studied traits, as estimated based on two-way ANOVA. (**b**) Statistically significant effects (*p* < 0.05) of sheep population (fat-tailed or thin-tailed sheep), sex and their interaction on studied traits, as estimated based on two-way ANOVA.

**(a)**						
**Trait**	**Effect**	**Sum of Squares**	**df**	**Mean Square**	**F**	***p*** **-Value**
Wither height (m)	Sheep population	0.01	1	0.01	5.53	<0.05
	LWC	2.28	4	0.57	269.96	<0.001
	Sheep population × LWC	0.03	4	0.01	3.45	<0.01
Yellowness—b*	Sheep population × LWC	11.80	4	2.95	2.56	<0.05
Hue angle	Sheep population × LWC	0.07	4	0.02	4.63	<0.01
Meat springiness	Sheep population × LWC	0.14	4	0.03	3.77	<0.01
**(b)**						
**Trait**	**Effect**	**Sum of Squares**	**df**	**Mean Square**	**F**	***p*** **-Value**
Wither height (m)	Sex	0.06	1	0.06	4.97	<0.05
Lightness—L*	Sex	64.00	1	64.03	7.69	<0.01
Redness—a*	Sex	12.79	1	12.79	6.14	<0.05
	Sheep population × Sex	8.40	1	8.40	4.03	<0.05
Yellowness—b*	Sex	13.40	1	13.40	12.46	<0.001
Chroma	Sex	19.85	1	19.85	8.07	<0.01
	Sheep population × Sex	10.76	1	10.76	4.37	<0.05
Hue angle	Sex	0.04	1	0.04	9.39	<0.01

**Table 7:** (**a**)**.** Statistically significant effects (*p* < 0.05) of sheep population (fat-tailed or thin-tailed sheep), live-weight category (LWC) and their interaction on studied traits, as estimated by Scheirer–Ray–Hare tests. (**b**) Statistically significant effects (*p* < 0.05) of sheep population (fat-tailed or thin-tailed sheep), sex and their interaction on studied traits, as estimated by Scheirer–Ray–Hare tests.

**(a)**					
**Trait**	**Effect**	**Sum of Squares**	**df**	**H**	***p*-Value**
Live weight (kg)	LWC	1,142,662.00	4	231.27	<0.001
Carcass length (m)	LWC	787,969.00	4	192.88	<0.001
Hot carcass weight (kg)	LWC	1,093,735.00	4	221.39	<0.001
Carcass yield (%)	Sheep population	36,751.00	1	7.44	<0.01
	LWC	705,885.00	4	142.86	<0.001
Carcass pH	LWC	48,815.00	4	10.75	<0.05
	Sheep population × LWC	44,529.00	4	9.80	<0.05
Lightness—L*	LWC	32,178.00	4	38.23	<0.001
Redness—a*	Sheep population	3560.00	1	4.23	<0.05
Meat pH	LWC	13,493.00	4	16.07	<0.01
Meat cohesiveness	Sheep population × LWC	9767.00	4	11.84	<0.05
**(b)**					
**Trait**	**Effect**	**Sum of Squares**	**df**	**H**	***p*** **-Value**
Live weight (kg)	Sex	36,855.00	1	7.46	<0.01
Hot carcass weight (kg)	Sex	20,769.00	1	4.20	<0.05
Carcass yield (%)	Sheep population	36,751.00	1	7.44	<0.01
	Sex	93,536.00	1	18.93	<0.001
Muscle fiber minimum Feret’s diameter (μm)	Sex	2569.00	1	5.27	<0.05
Carcass pH	Sheep population × Sex	21,223.00	1	4.67	<0.05

## Data Availability

The data presented in this study are available on request from the corresponding author.

## Supplementary Materials

The following supporting information can be downloaded at: https://www.mdpi.com/article/10.3390/ani12151998/s1, Table S1a: Effects of sheep population (fat-tailed or thin-tailed sheep), live-weight category (LWC—% of mature live weight) and their interaction on studied traits, as estimated based on two-way ANOVA; Table S1b: Effects of sheep population (fat-tailed or thin-tailed sheep), sex and their interaction on studied traits, as estimated based on two-way ANOVA; Table S2a: Effects of sheep population (fat-tailed or thin-tailed sheep), live-weight category (LWC) and their interaction on studied traits, as estimated by Scheirer–Ray–Hare tests; Table S2b: Effects of sheep population (fat-tailed or thin-tailed sheep), sex and their interaction on studied traits, as estimated by Scheirer–Ray–Hare tests.
