# Supplementary material for: A Comprehensive Study of the Quality of Fat-Tailed Sheep Carcasses in Greece
Source: Animals (Basel). 2022 Aug 7;12(15):1998. doi: 10.3390/ani12151998 (PMC9367314; doi:10.3390/ani12151998)
Supplement: Supplementary file 1 [file animals-12-01998-s001.zip › animals-1833618-supplementary.pdf]

Table S1a. Effects of sheep population (fat-tailed or thin-tailed sheep), live-weight category (LWC—% of mature live weight) and their interaction on studied traits, as estimated based on two-way ANOVA.

Trait	Effect	Sum of squares	df	Mean Square	F	P-value
Wither height (m)	Sheep population	0.01	1	0.01	5.53	<0.05
	LWC	2.28	4	0.57	269.96	<0.001
	Sheep population × LWC	0.03	4	0.01	3.45	<0.01
Yellowness - b*	Sheep population	0.03	1	0.03	0.03	0.871
	LWC	3.53	4	0.88	0.77	0.549
	Sheep population × LWC	11.80	4	2.95	2.56	<0.05
Hue angle	Sheep population	0.00	1	0.00	1.04	0.311
	LWC	0.02	4	0.01	1.65	0.170
	Sheep population × LWC	0.07	4	0.02	4.63	<0.01
Meat hardness 1 (g)	Sheep population	1,093,742.00	1	1,093,742.00	2.01	0.160
	LWC	3,357,320.00	4	839,330.00	1.54	0.197
	Sheep population × LWC	3,412,780.00	4	853,195.00	1.57	0.190
Meat hardness 2 (g)	Sheep population	752,817.00	1	752,817.00	2.46	0.120
	LWC	1,867,845.00	4	466,961.00	1.53	0.202
	Sheep population × LWC	1,826,880.00	4	456,720.00	1.49	0.211
Meat springiness	Sheep population	0.00	1	0.00	0.45	0.506
	LWC	0.07	4	0.02	2.01	0.100
	Sheep population × LWC	0.14	4	0.03	3.77	<0.01
Meat chewiness	Sheep population	260,012.00	1	260,012.00	3.58	0.062
	LWC	568,876.00	4	142,219.00	1.96	0.108
	Sheep population × LWC	552,548.00	4	138,137.00	1.90	0.117

Table S1b. Effects of sheep population (fat-tailed or thin-tailed sheep), sex and their interaction on studied traits, as estimated based on two-way ANOVA.

Trait	Effect	Sum of squares	df	Mean Square	F	P-value
Wither height (m)	Sheep population	0.01	1	0.01	1.02	0.314
	Sex	0.06	1	0.06	4.97	<0.05
	Sheep population × Sex	0.00	1	0.00	0.29	0.594
Lightness - L*	Sheep population	13.40	1	13.40	1.61	0.208
	Sex	64.00	1	64.03	7.69	<0.01
	Sheep population × Sex	1.30	1	1.29	0.16	0.695
Redness - a*	Sheep population	4.01	1	4.01	1.93	0.169
	Sex	12.79	1	12.79	6.14	<0.05
	Sheep population × Sex	8.40	1	8.40	4.03	<0.05
Yellowness - b*	Sheep population	0.03	1	0.03	0.03	0.866
	Sex	13.40	1	13.40	12.46	<0.001

	Sheep population × Sex	2.24	1	2.24	2.08	0.153
**Chroma**	Sheep population	3.65	1	3.65	1.49	0.226
	Sex	19.85	1	19.85	8.07	<0.01
	Sheep population × Sex	10.76	1	10.76	4.37	<0.05
**Hue angle**	Sheep population	0.00	1	0.00	0.95	0.331
	Sex	0.04	1	0.04	9.39	<0.01
	Sheep population × Sex	0.00	1	0.00	0.31	0.577
**Meat springiness**	Sheep population	0.00	1	0.00	0.38	0.540
	Sex	0.00	1	0.00	0.01	0.914
	Sheep population × Sex	0.00	1	0.00	0.05	0.833
**Meat cohesiveness**	Sheep population	0.00	1	0.00	0.63	0.428
	Sex	0.00	1	0.00	0.00	0.973
	Sheep population × Sex	0.01	1	0.01	3.10	0.082

Table S2a. Effects of sheep population (fat-tailed or thin-tailed sheep), live-weight category (LWC) and their interaction on studied traits, as estimated by Scheirer–Ray–Hare tests.

Trait	Effect	Sum of squares	df	H	P-value
**Live weight (kg)**	Sheep population	502.00	1	0.10	0.750
	LWC	1,142,662.00	4	231.27	<0.001
	Sheep population × LWC	1,763.00	4	0.36	0.986
**Carcass length (m)**	Sheep population	3,205.00	1	0.78	0.376
	LWC	787,969.00	4	192.88	<0.001
	Sheep population × LWC	3,916.00	4	0.96	0.916
**Hot carcass weight (kg)**	Sheep population	786.00	1	0.16	0.690
	LWC	1,093,735.00	4	221.39	<0.001
	Sheep population × LWC	4,598.00	4	0.93	0.920
**Carcass yield (%)**	Sheep population	36,751.00	1	7.44	<0.01
	LWC	705,885.00	4	142.86	<0.001
	Sheep population × LWC	18,032.00	4	3.65	0.456
**Carcass pH**	Sheep population	5,824.00	1	1.28	0.257
	LWC	48,815.00	4	10.75	<0.05
	Sheep population × LWC	44,529.00	4	9.80	<0.05
**Lightness - L***	Sheep population	816.00	1	0.97	0.325
	LWC	32,178.00	4	38.23	<0.001
	Sheep population × LWC	1,088.00	4	1.29	0.863
**Redness - a***	Sheep population	3,560.00	1	4.23	<0.05
	LWC	2,439.00	4	2.90	0.575
	Sheep population × LWC	1,927.00	4	2.29	0.683
**Chroma**	Sheep population	2,464.00	1	2.93	0.087
	LWC	2,375.00	4	2.82	0.588

	Sheep population × LWC	2,061.00	4	2.45	0.654
**Meat pH**	Sheep population	14.00	1	0.02	0.897
	LWC	13,493.00	4	16.07	<0.01
	Sheep population × LWC	7,309.00	4	8.70	0.069
**Meat cohesiveness**	Sheep population	92.00	1	0.11	0.738
	LWC	1,130.00	4	1.37	0.849
	Sheep population × LWC	9,767.00	4	11.84	<0.05

Table S2b. Effects of sheep population (fat-tailed or thin-tailed sheep), sex and their interaction on studied traits, as estimated by Scheirer–Ray–Hare tests.

Trait	Effect	Sum of squares	df	H	P-value
**Live weight (kg)**	Sheep population	502.00	1	0.10	0.750
	Sex	36,855.00	1	7.46	<0.01
	Sheep population × Sex	654.00	1	0.13	0.716
**Carcass length (m)**	Sheep population	3,205.00	1	0.78	0.376
	Sex	13,545.00	1	3.32	0.069
	Sheep population × Sex	46.00	1	0.01	0.916
**Hot carcass weight (kg)**	Sheep population	786.00	1	0.16	0.690
	Sex	20,769.00	1	4.20	<0.05
	Sheep population × Sex	663.00	1	0.13	0.714
**Carcass yield (%)**	Sheep population	36,751.00	1	7.44	<0.01
	Sex	93,536.00	1	18.93	<0.001
	Sheep population × Sex	197.00	1	0.04	0.842
**Muscle fiber minimum Feret's diameter (μm)**	Sheep population	490.00	1	1.01	0.316
	Sex	2,569.00	1	5.27	<0.05
	Sheep population × Sex	804.00	1	1.65	0.199
**Carcass pH**	Sheep population	5,824.00	1	1.28	0.257
	Sex	700.00	1	0.15	0.695
	Sheep population × Sex	21,223.00	1	4.67	<0.05
**Meat pH**	Sheep population	14.00	1	0.02	0.897
	Sex	2,670.00	1	3.18	0.075
	Sheep population × Sex	128.00	1	0.15	0.696
**Meat hardness 1 (g)**	Sheep population	1,801.00	1	2.18	0.140
	Sex	50.00	1	0.06	0.805
	Sheep population × Sex	292.00	1	0.35	0.552
**Meat hardness 2 (g)**	Sheep population	1,889.00	1	2.29	0.130
	Sex	45.00	1	0.06	0.814
	Sheep population × Sex	385.00	1	0.47	0.494
**Meat chewiness**	Sheep population	2,241.00	1	2.72	0.099
	Sex	173.00	1	0.21	0.647
	Sheep population × Sex	752.00	1	0.91	0.340

**Meat moisture (%)**	Sheep population	9.60	1	0.06	0.806
	Sex	114.50	1	0.73	0.394
	Sheep population × Sex	285.70	1	1.81	0.178
**Meat lipid content (%)**	Sheep population	224.80	1	1.43	0.232
	Sex	307.50	1	1.95	0.163
	Sheep population × Sex	16.40	1	0.10	0.747
**Meat protein content (%)**	Sheep population	13.80	1	0.09	0.768
	Sex	94.20	1	0.60	0.439
	Sheep population × Sex	76.80	1	0.49	0.485
